# Increasing health inequalities between women in and out of work - the impact of recession or policy change? A repeated cross-sectional study in Stockholm county, 2006 and 2010

**DOI:** 10.1186/1475-9276-13-51

**Published:** 2014-07-25

**Authors:** Sandra Blomqvist, Bo Burström, Mona C Backhans

**Affiliations:** 1 Public Health Sciences, Karolinska Institutet, 171 77 Stockholm, Sweden; 2 Centre for Epidemiology and Community Medicine, Stockholm County Council, 171 29 Solna, Sweden

**Keywords:** Labour market position, Social insurance, GHQ12, Limiting longstanding illness, Repeated cross-sectional survey

## Abstract

**Introduction:**

The social insurance system in Sweden underwent extensive change between 2006 and 2010, with the overall aim of making people enter the labour market. At the same time, economic recession hit Sweden. Previous studies suggest that the economic recession particularly affected women. In light of these changes, the aim of this study is to investigate whether health inequalities between employed women and groups outside the labour market changed between 2006 and 2010. A second aim is to examine the explanatory weight of socio-demographic factors vs social and economic conditions.

**Methods:**

Data consists of the Stockholm Public Health Surveys (SPHS) for 2006 and 2010. Women aged 18–64 were studied. Through logistic regression, levels of mental distress and limiting longstanding illness (LLI), were compared between four labour market groups; employed and unemployed, sickness absentees and disability pension recipients, at the two time points.

**Results:**

Mental distress increased among women in all four labour market groups between 2006 and 2010. Differences in mental distress between those employed and groups outside the labour market also increased. These were explained primarily by social and economic conditions. Levels of LLI were unchanged except among the unemployed. The difference in LLI between the unemployed and the employed was mostly explained by social and economic conditions. In the other groups socio-demographic factors were more salient. For both health outcomes, the weight of social and economic conditions had increased in 2010 compared to 2006.

**Conclusions:**

Results indicate that levels of mental distress increased in all groups, but more so among groups outside the labour market, possibly due to stricter eligibility criteria and lower benefit levels, which particularly affected their social and economic conditions.

## Introduction

Between 2006 and 2010 the Swedish social insurance system underwent several modifications, including the introduction of time limits for receiving sickness benefit, raised fees for membership in unemployment insurance funds and the introduction of a working tax credit. In general the reforms resulted in lower compensation levels and stricter eligibility criteria. During this period Sweden was also hit by the economic recession which started in 2008. The unemployment level increased and more people were in need of compensation from the social insurance system.

Research on health consequences of the business cycle has shown that mental health is negatively affected by economic recession [1]. In addition groups outside the labour market are more adversely affected by recession, in social and economic terms, than those in the labour market [2-5]. Previous Swedish studies have found a stronger association between unemployment and negative health outcomes among women compared to men, and that women to a higher extent than men report lower health status during recession [6,7]. Furthermore, in a study on employment consequences in different socioeconomic groups in Sweden of the 1990’s recession, women with limiting longstanding illness and with low socioeconomic status were particularly hard hit [8].

In light of these findings, this study focuses on women. The aim is to investigate whether health inequalities between employed women and those not in work (unemployed, on sick leave or with disability pension) has increased in 2010 compared to 2006, regarding limiting longstanding illness and mental distress. A second aim is to examine whether inequalities are explained by socio-demographic factors or social and economic conditions in the groups.

### The social insurance system in Sweden

According to Swedish law a person is entitled to sickness benefit in case of illness and reduced work ability. Disability pension is granted when the ability to work is considered to be reduced by at least 25% for one year or more (Law 1962:381 on public insurance). The purpose of the insurance is to provide a financial substitute to employment income in case of illness [9]. There is a maximum amount of compensation that can be received and a lowest level of income required to receive any compensation [10]. Until July 2008 there was no time limit for receiving sickness benefit. The full sickness benefit was 80% of the income and disbursed by the Swedish social insurance agency, except for the first two weeks which is paid by the employer [11].

Unemployment insurance is divided into two tiers. The basic benefit is a flat-rate compensation for those who meet the criteria for compensation but are not a part of an unemployment insurance fund. The other form is an income-related benefit requiring membership in an unemployment insurance fund and fulfilled conditions of work during the membership period. The benefit is based on the applicant’s former income but with a maximum and a minimum amount that can be obtained. [12] Unemployment benefits in 2005 were 80% replacement rate below an income ceiling, and the qualifying work time was 70 h/month during 6 months.

### Changes in social insurance between 2006 and 2010

When the right-wing government came into power in 2006, it immediately introduced a number of reforms within sickness and unemployment insurance. The policy changes are listed in Table 1. One of the main changes in sickness insurance during the period was the introduction of sick-listing guidelines to provide doctors with recommendations of suitable sick-listing times for certain diagnoses. In addition the so called rehabilitation chain was introduced in July 2008. The reform inferred a more restrictive approach in terms of a time limit of compensation and dates for assessment of work ability. A main feature of this reform is the obligation of the insured person to take a job anywhere in Sweden, not only in the area where the person is resident, if the assessment established that the person was not eligible for compensation from sickness insurance. The reform also implied decreased compensation the longer the period of sick spell proceeded. Also stricter criteria for disability pension were introduced. It is now only granted if the capacity to work is considered to be reduced for the foreseeable future. On the other hand it was made possible for disability pension recipients to try out work without losing their compensation, making it easier for this group to return to work.

**Table 1:** Policy changes 2006–2010 in unemployment and sickness insurance

**Date**	**Sickness insurance**	**Date**	**Unemployment insurance**
2006-07	The maximum level of sickness benefit qualifying income is raised from 7.5 to 10 times the price base amount.	2007-01	Raised fee for membership in unemployment insurance funds.
2007	Introduction of sick-listing guidelines. Recommendations of suitable sick-listing times for certain diagnoses.	2007-01	Stricter working conditions for entitlement to compensation, from 70 h/month during 6 months to 80/month during 6 months.
2007-01	Lowered compensation level from 10 times the price base amount to 7.5.	2007-01	Prolonged time frame from which the compensation is based on. From a compensation based on 6 months in a frame of 12 months to the whole frame of 12 months.
2008-07	Introduction of the rehabilitation chain. Stricter criteria and time limits for compensation and a reduction of compensation the longer the period of sick spell proceeds. Specific dates for assessment of work ability.	2007-01	The possibility to have compensation based on study time is removed.
2008-07	More stringent eligibility for disability pension.	2007-03	Time limits of compensation are introduced where the compensation is reduced as the unemployment proceeds.
2008-07	A person has a right to take leave because of illness to try another job.	2007-07	Prolonged period of compensation is taken away; maximum of compensation is during 300 days.
2008-12	Among disability pension recipients, compensation from insurance is reduced in connection with income from work.	2007-10	Days with unemployment benefit and/or activity grant is summed together meaning a maximum of days (300) is compensated regardless which type of compensation.
2009-01	A person is no longer entitled to let the compensation from the Social Insurance Agency rest for 3 month, when trying out work. Instead a smaller amount is paid monthly.	2007-12	Youth job program is introduced for those aged 16-24, who have been unemployed for at least 3 months and registered as actively seeking job during this time.
2009-07	Those who receive an activity grant are seen as unemployed and are transferred to the unemployment insurance fund if they are in an Active Labour Market Program (ALMP).	2008-04	Limitation of unemployment benefit when working part time.
2010-01	Those who have received the maximum number of days with sickness benefit or a time-limited disability pension are offered to participate in ALMP and receive activity compensation.	2008-07	Number of waiting days is raised from 5 to 7.
2010-01	The assessment of the person’s ability to work towards the whole labor market can be postponed if it is not considered suitable.	2009-07	More lenient criteria for unemployment insurance membership.
2010-01	Due to serious illness, the period of sickness benefit at 80% replacement rate can be prolonged after 364 days.	2010-01	People with maximum days of sickness benefit or time-limited disability pension can obtain unemployment benefit if certain criteria are fulfilled.
2010-07	For the self-employed a more generous calculation of the sickness qualified income and several alternatives for the number of waiting days is introduced.	2010-01	12 month of membership for entitlement to unemployment benefit based on earlier income.

Reforms in unemployment insurance during the studied period consist of more stringent criteria for entitlement to the income-related benefit as well as higher demands on the job-seeker to actively seek work in the whole country. Also the fee for membership in unemployment insurance funds was raised. Subsequently, time limits and reduced benefit levels at prolonged unemployment were incorporated into unemployment insurance [13] with a maximum of 300 days with benefits. In 2007, the possibility to have compensation based on study time was removed. Another reform during the period that was introduced in four stages is a form of tax reduction (working tax credit), to enhance the economic incentives to work. The tax reduction only concerns income from work meaning that employed in contrast to benefit recipients get to keep a larger part of their wage due to reduced local income tax.

**Figure 1:**
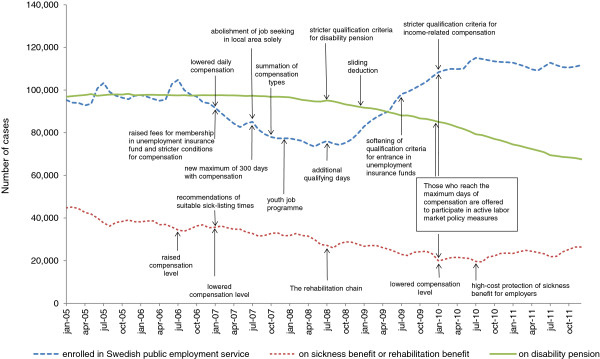
Number of cases of registered unemployed, on sickness or rehabilitation benefits, and disability pension in 2005–2011 in Stockholm county.

The main purpose of these reforms was to ‘make work pay’, thus creating incentives for people out of work to return to work. However, critics of the reforms say that creating obstacles to enter the insurance system implies a hollowing-out of the traditionally inclusive and generous benefit system, where those most removed from the labour market due to lack of experience or low education, are effectively barred from income-related benefits. For those with illness, being too well for the insurance but too ill for the demands of employers may simply mean a change of insurance type, from sickness to unemployment benefits, with often less generous benefits due to the ceiling effect.Figure 1 displays the number of people registered as unemployed at the Swedish public employment service and the number of people receiving sickness benefit or disability pension from the Swedish social insurance agency during 2005–2011 in Stockholm county. The figure shows monthly statistics for Stockholm County and arrows illustrate when changes in the social insurance system were introduced.

### Previous studies

The association between health and labour market position is usually described in terms of effects of selection and effects of exposure [2,6,14]. The relative weight of selection and exposure is likely to differ between groups, for example people on disability pension are by definition ill. It is also probable that selection and exposure play different roles depending on the health outcome studied.

Mental distress, depression, a low level of well-being as well as low self-esteem is more common among the unemployed and sick listed than among the employed [4,15-17]. Part of this may be explained by financial strain and social isolation [3-5]. Interviewed women with a long-term sickness absence reported that they felt isolated and lonely as the sick spell proceeded [18]. Furthermore the sick spell gave them time to reflect on their pain which had an intensifying effect and created a vicious circle with further isolation and distress. Financial strain and loneliness have also been shown to be mediating factors of low self-assessed health among the unemployed [19]. Other studies have found that the unemployed are more likely to report unmet care needs than the employed [20].

The demand for labour varies depending on the business cycle. When the economy is flourishing and unemployment is low, people with less serious health problems are more likely to work. Thus, health selection into unemployment is strengthened. During boom years sickness absence levels tend to increase [21-23]. This has been explained by a higher proportion of people in poor health in the labour force and consequently more people at risk of being sick-listed [24]. In times of recession, sickness absence levels decrease. This may be due to a combination of increased selection [25] and a disciplining effect among people in employment affecting the likelihood of a person taking sick leave.

Health selection into groups inside and outside the labour market can partly be explained by the strictness of eligibility criteria for social insurance. A system with a stricter set of criteria leads to a lower number of people being eligible for compensation and in turn an increased selection of less healthy people out of labour [16]. Research has also shown that economic incentives to work influence the extent to which people take sick leave, as well as time in unemployment [21,26,27].

### Hypothetical effects on health of policy change

Through an overall assessment of the changes in the social insurance system and based on the research presented above, hypothetical effects on health of policy change among people on sick leave/disability pension and in unemployment are presented below.

– Sickness leave/disability pension recipients: Due to changes in sickness insurance it is plausible to expect a poorer health status among those on sick leave since only the “truly” ill will qualify. This ought to be the case also for those on disability pension. These changes are both expected and intended.

– Unemployed: It is plausible that the unemployed will be relatively worse off as well. Due to stricter eligibility criteria people who earlier qualified for the sickness insurance may be found among the unemployed. These changes may be described as unintended consequences of policy change.

– All groups: stricter criteria to receive income-related benefits, as well as decreased compensation levels and a decrease of the maximum number of days with compensation is likely to lead to higher levels of mental distress. The effects on LLI are probably minor in the short term.

It is also possible that the expected changes are affected by the business cycle. For example, one effect of the economic recession could be that people who are not ill enough for sickness insurance are deemed too ill by employers. In a more favourable economic situation, it is more likely that these people would have become employed.

### Data and methods

#### Study population

The study used a repeated cross-sectional design. The data consist of the Stockholm Public Health Surveys for 2006 and 2010. The study population consists of an area-stratified random sample of the population of Stockholm county aged 18–84 years. However, this study is restricted to those 18–64 years. In 2006 the sample in this age range consisted of 47 624 persons. The survey was answered by 27 994 people (58.8% response rate). In 2010 the sample consisted of 44 364 persons and there were 22 639 respondents (51% response rate). Survey data was complemented with information from the register Longitudinal integration database for health insurance and labour market studies (LISA). Ethical approval was obtained by the Stockholm Regional Ethical Review Board (Dnr 2010/1879 − 31/5; Dnr 2007/545 − 31).

**Figure 2:**
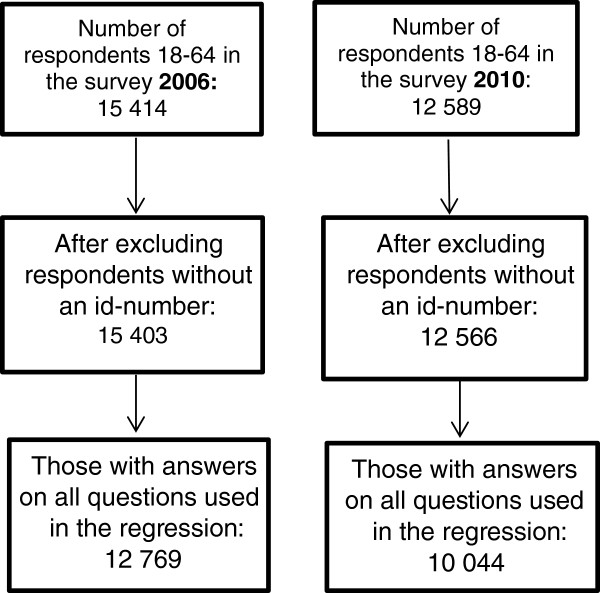
Flowchart of participants in 2006 and 2010.

The survey suffers from response bias meaning that respondents have a higher average age, higher income, and a higher educational level than the whole population. Calibration weights have been constructed by Statistics Sweden to compensate for selective drop-out and these weights were used in the analysis [28].Figure 2 illustrates the selection of respondents throughout the analysis. The final number of respondents was 12 769 in 2006 and 10 044 in 2010.

### Main predictor

Labour market position is our main predictor. The measure is based on one survey question: What is your primary activity right now? The question was categorized in the following way (see Table 2):

**Table 2:** The operationalization of labour market position

**Labor market position**	**Alternative in the questionnaire**
	Permanently employed
Employed	Temporary employed
	Self employed
	On leave or parental leave
Unemployed	Job seeking or in ALMP
Sick leave	Sickness absent more than 30 days
Disability pension	On disability pension

Respondents who were retired, students, keeping house or ‘other’ were excluded. In the survey of 2006 it was possible to mark several alternative activities while in 2010 only one main alternative was allowed. This was handled in the following way: if the respondent had marked employed, he/she was always considered employed. If the respondent had a combination of unemployed, on sick leave and/or disability pension the respondent was excluded from the analysis. Only a small number, 169 of 12,769, had filled in any of these combinations and their exclusion should not affect results.

### Health outcomes

Two health indicators from the questionnaire were used. Mental distress was measured using the General Health Questionnaire (GHQ12). GHQ12 is one of the most frequently used psychiatric screening instruments, with the aim of discriminating between those with and without minor psychiatric disorders. The index was coded into a dichotomous variable with a cut off at three or higher. Although a cut-off score of 3/4 is sometimes found to be optimal, we chose 2/3 as this is recommended in low prevalence populations [29,30].

The other indicator was limiting long-standing illness (LLI) defined as a longstanding health problem which is causing limited work ability or limits the respondent in other daily activities. These outcomes were chosen as they represent two distinct dimensions of health. Both measures are widely used in research, validated and considered suitable measures of ill health [31,32].

### Confounders and mediators

We controlled for a number of socio-demographic factors; age, educational level, country of origin and cohabiting status. Age was categorized into 18–29, 30–49 and 50–64 years. Educational level was categorized into primary/lower secondary, upper secondary and tertiary education. Country of origin was dichotomized into Swedish or foreign-born. Cohabitation status was dichotomized based on whether the respondent was or was not living with a partner, parent, sibling or another adult whether with or without children. This categorization was made with an economic perspective and with a presumption of shared household expenditures. However, in cases where respondents are living with parents or siblings it is possible that expenditures are not shared.

In a second step, we controlled for social and economic conditions that may mediate the impact of labour market position on mental distress and LLI. Whether the respondent has participated more or less regularly in social activities during the past 12 months, if the respondent has received social assistance or abstained from medical care, dental care or medicine for economic reasons constitute the social and economic conditions. Initially relative poverty was included, measured as having 60% or less than the median disposable income. This variable was not significant in any analysis and it was therefore excluded. Social assistance was a predictor for LLI in 2006 when included on its own, but not in a model including the main predictor.

### Statistical analysis

Statistical analysis system (SAS) 9.3 was used. Odds ratios (OR) for differences between employed, unemployed, those on sick leave and disability pension for the two health outcomes in 2010 and in 2006 were analysed using logistic regression. Employed persons were used as reference group.

## Results

### Descriptive analysis

Table 3 shows the distribution of ill health, socio-demographic factors and social and economic conditions in the different labour market groups. The proportion of women with mental distress generally increased between 2006 and 2010. The prevalence was especially high among those on sick leave. Furthermore the difference in mental distress between employed women and the other three groups increased. The table also shows that there were small changes in LLI between 2010 and 2006. The most distinct increase was seen for unemployed women.

**Table 3:** Health and social characteristics by labor market position

	**2006**			
	**Employed**	**Unemployed**	**Sick leave**	**Disability pension**
**2006**	**n = 11,557 (83%.7)**	**n = 699 (6%.0)**	**n = 394 (2%.8)**	**n = 980 (7%.5)**
**Health**				
Mental distress	20.1	39.4	55.9	33.7
Limiting longstanding illness	14.6	24.3	83.7	90.6
**Socio-demographic factors**				
Average age	40.9	36.5	44.1	54.1
Primary/lower secondary	12.8	33.0	18.5	29.7
Upper secondary	59.4	51.3	64.7	59.5
Tertiary	27.8	15.7	16.8	10.8
Born abroad	19.9	37.4	31.1	32.5
Living alone	24.8	23.9	35.9	40.4
**Economic and social conditions**				
Abstained from care due to economic reasons	21.7	41.7	38.7	35.6
Not participated in activities in the past 12 months	40.3	49.9	56.8	54.9
Received social assistance	2.8	14.3	11.4	6.9
Received disability pension	1.1	2.3	7.4	86.2
Received unemployment benefit	7.4	31.4	7.4	2.7
Received sickness benefit	8.4	13.4	54.9	20.9
	**Employed**	**Unemployed**	**Sick leave**	**Disability pension**
**2010**	n = 9,543 (88**%**.2)	n = 402 (4**%**.7)	n = 161 (1**%**.7)	n = 522 (5**%**.3)
**Health**				
Mental distress	22.7	44.9	64.8	39.2
Limiting longstanding illness	15.3	27.8	83.8	91.2
**Socio-demographic factors**				
Average age	41.8	38.5	46.0	53.6
Primary/lower secondary	10.7	30.6	24.8	33.5
Upper secondary	55.4	44.0	55.2	58.1
Tertiary	33.9	25.4	20.0	8.4
Born abroad	23.7	39.4	35.1	37.1
Living alone	21.4	19.2	30.9	48.0
**Economic and social conditions**				
Abstained from care due to economic reasons	17.6	43.3	43.5	43.5
Not participated in activities in the past 12 months	38.8	54.0	56.4	57.8
Received social assistance	2.0	13.8	21.9	5.5
Received disability pension	2.4	6.2	17.5	94.6
Received unemployment benefit	4.4	19.7	1.7	2.1
Received sickness benefit	7.4	9,7	46.2	8.2

There was a marked increase of sick-listed women receiving social assistance (from 11% to 22%) and a decrease in the prevalence receiving sickness insurance benefits. In all other groups there was a small decrease in social assistance. The proportion with unmet care needs due to economic reasons increased from 2006 to 2010 in all groups except among the employed. The greatest increase was found among those with disability pension (from 36% to 44%). Regarding regular participation in social activities, the proportion decreased among the employed, while there was a small increase among the unemployed and disability pension recipients. Furthermore, those on disability pension had the highest prevalence living alone while the unemployed had the lowest prevalence.

### Logistic regression

Table 4 shows the difference in mental distress and LLI among the unemployed, sickness absentees and disability pension recipients compared to the employed group in 2006 and 2010. All groups outside the labour market had higher levels of mental distress compared to employed women. Those on sick leave have the highest risk in both years, and the difference to the employed group has increased in 2010 compared to 2006. Socioeconomic and demographic factors were adjusted for in model 1. Apart from unemployed women, it is apparent that the socio-demographic factors did not explain differences in mental distress relative to employed women. The unemployed have a lower average age than the other groups, which could be one reason why the unemployed women differ from the rest. In contrast to the socio-demographic factors, mental distress is partly explained by social and economic conditions among women outside the labour market, especially among sickness absentees (model 2). Among women on disability pension or on sick leave social and economic conditions explain more of the difference in mental distress compared to the employed in 2010 compared to 2006.

**Table 4:** The association between labour market position and health in 2006 and 2010

**Mental distress**	**Crude**		**Model 1**		**Model 2**		**Full model**	
	**OR**	**CI**	**OR**	**CI**	**OR**	**CI**	**OR**	**CI**
**2006**								
Employed	1		1		1		1	
Unemployed	2.65	(2.17-3.23)	2.38	(1.95-2.92)	2.14	(1.73-2.63)	2.10	(1.70-2.58)
Sick leave	4.64	(3.62-5.96)	5.14	(3.95-6.70)	4.01	(3.11-5.18)	4.60	(3.53-6.01)
Disability pension	1.92	(1.62-2.29)	2.73	(2.26-3.31)	1.66	(1.39-1.98)	2.38	(1.96-2.89)
**2010**								
Employed	1		1		1		1	
Unemployed	2.81	(2.20-3.58)	2.66	(2.07-3.42)	2.27	(1.75-2.94)	2.23	(1.75-2.96)
Sick leave	6.54	(4.36-9.82)	7.12	(4.65-10.90)	5.47	(3.55-8.43)	6.19	(3.98-9.65)
Disability pension	2.29	(1.84-2.86)	2.97	(2.34-3.77)	1.75	(1.39-2.20)	2.37	(1.86-3.03)
**LLI**	**Crude**		**Model 1**		**Model 2**		**Full model**	
	**OR**	**CI**	**OR**	**CI**	**OR**	**CI**	**OR**	**CI**
**2006**								
Employed	1		1		1		1	
Unemployed	2.02	(1.62-2.52)	1.93	(1.54-2.42)	1.63	(1.31-2.04)	1.64	(1.30-2.06)
Sick leave	30.89	(21.54-44.28)	28.50	(19.84-41.00)	28.07	(19.41-40.61)	26.26	(18.16-37.96)
Disability pension	56.28	(42.79-74.00)	44.03	(33.35-58.15)	53.40	(40.65-70.14)	40.98	(31.03-54.12)
**2010**								
Employed	1		1		1		1	
Unemployed	2.25	(1.72-2.95)	2.25	(1.71-2.95)	1.80	(1.38-2.35)	1.88	(1.40-2.53)
Sick leave	29.86	(17.23-51.76)	26.10	(15.13-45.62)	25.31	(14.87-43.10)	26.02	(14.12-47.97)
Disability pension	58.37	(39.37-86.53)	37.10	(25.21-54.62)	52.47	(35.66-77.21)	38.06	(24.69-58.68)

Odds ratios and confidence intervals.

Model 1 adjusted for sex, age, education, country of origin, single household. Model 2 adjusted for regular participation in social activities, social assistance, abstained from care. Full model adjusted for all of the above.

Groups outside the labour market also have a higher risk of having LLI than women in the labour market (Table 4). Differences in relation to the employed was quite stable between 2006 and 2010. Among those on disability pension, socio-demographic factors explained most of the difference in LLI between this group and employed women, and these factors are also of greater importance in 2010 compared to 2006. For the unemployed and those on sickness absence, social and economic conditions are somewhat more important than socio-demographic factors.

## Discussion

We hypothesised that the modifications in the social insurance system in terms of stricter eligibility criteria, lower compensation levels and shorter periods with compensation could lead to higher levels of mental distress among groups outside the labour market, but that effects on LLI in the short term would be minor. In accordance with this the results showed that differences in mental distress between the employed and the other groups increased over time, while levels of LLI were generally unchanged. However, pooled analyses where all groups/years were simultaneously compared show that these changes are not statistically significant, thus we should talk more of tendencies than definitive differences.

Whether the increased health inequalities are caused by health selection or reflect direct effects of changes in the social insurance system is hard to tell. However, the analysis showed that social and economic conditions were more important as explanatory factors in 2010 compared to 2006. This is in accordance with previous research showing that groups outside the labour market have been more adversely affected [2-5], and could be a reflection of changes in the insurance system, that have led to financial difficulties, and increased levels of stress due to uncertainty about future eligibility. An indication of these policy changes are the growing proportion of women on sick leave who received social assistance in 2010 compared to 2006, while a smaller proportion received compensation from sickness insurance. Moreover, a smaller proportion among the unemployed received unemployment benefits. The introduction of a tax reduction of income from work could also be a contributing factor to the increased health inequalities between employed and those outside the labour market. A recent study using EU SILC data [33] found a nearly doubled rate of being at risk of poverty among persons with LLI in Sweden from 2005 to 2010, while a similar increase was not observed in Denmark or the United Kingdom. An alternative explanation is that the poor economy has led to longer times spent out of work and poorer prospects for the future, leading to depression and social isolation. It is likely that policy change and recession may interact to exacerbate negative effects.

We expected that health selection would increase partly due to policy change, for example qualification requirements for sickness insurance, as the stricter criteria leads to stronger health selection into sickness benefit and disability pension. Also a spill-over effect in the unemployed group was anticipated where people who earlier received compensation from the sickness insurance now are referred to unemployment. Other factors that could increase health selection are effects of the economic recession. In times of recession sickness absence tends to decrease, and health selection into sickness absence is strengthened [23]. Furthermore when the economic activity is low, (positive) health selection into employment is also likely to increase. Thus it is possible that part of the increasing differences in mental distress between those outside the labour market and those in labour is due to increasing health selection. However, as levels of LLI, which is likely to reflect more of health selection and less of direct effects, did not change in these groups, health selection seems like a less likely candidate than direct effects of policy change. As mental distress is a more immediate outcome than long-standing limiting illness, it is also likely that health effects emerge sooner. Thus, a certain time lag is plausible before policy change is reflected in chronic conditions among those outside the labour market.

### Strengths and limitations

The study design does not make it possible to attribute specific changes in social insurance to changes in health in different labour market groups. As mentioned, it is also likely to expect a certain time lag before changes in the social insurance system are reflected in levels of health among those outside the labour market. For example, some of the policy changes are close to the time of measurement in 2010. Decreasing response rates between time points means that more individuals with low education, low income and with a lower age are non-responders in 2010 compared to 2006 [34]. Although calibration weights have been constructed to deal with this problem, it may mean that changes between time points are underestimated.

The main predictor used in the study is the four labour market positions where unemployed, sickness absentees and disability pension recipients are compared to the employed. The classification into these groups is based on what the respondents themselves reported in the survey as their main activity. The group of young people not enrolled in employment, education or training (NEET) has increased during the past years and is highly affected by the consequences of being outside of the labour market. Furthermore it is likely that NEET will increase further due to stricter eligibility criteria. Therefore, a strength in this study is the use of self-reported activity status where this group is captured.

Given the more favourable labour market situation in Stockholm county, the results reported here may well be better than those for the whole country, as it is likely that time in unemployment or on sick leave is generally shorter. Due to contextual differences between sparsely populated areas and urban areas the results should only be generalised to similar regions. However the experience of being unemployed or on sick leave may be comparable both within Sweden and in countries with a similar welfare system.

## Conclusion

The results show clear, although not significant, tendencies of increased inequalities in mental distress between women in and out of the labour market between 2006 and 2010. Differences in mental health were mostly explained by social and economic conditions rather than socio-demographic factors. These results point to possible effects of social insurance policy change between 2006 and 2010, in addition to adverse effects of the economic recession. Given continuously high unemployment and increasing sickness absence, it is possible that health inequalities between the employed and excluded groups have increased further. Therefore, these groups should be monitored using more recent data.

## Competing interests

The authors declare that they have no competing interests.

## Authors’ contributions

SB Performed all data analysis, prepared the tables and figures, and was mainly responsible for the main manuscript text. BB Was involved in the study design and choice of focus. Read and approved the final manuscript, and contributed to the discussion and interpretation of results. MB Took part in and guided the analysis stage, read and discussed different versions of the manuscript and contributed to the main manuscript text. All authors read and approved the final manuscript.
